# Quercetin reduces obesity-induced hepatosteatosis by enhancing mitochondrial oxidative metabolism via heme oxygenase-1

**DOI:** 10.1186/s12986-015-0030-5

**Published:** 2015-10-06

**Authors:** Chu-Sook Kim, Yoonhee Kwon, Suck-Young Choe, Sun-Myung Hong, Hoon Yoo, Tsuyoshi Goto, Teruo Kawada, Hye-Seon Choi, Yeonsoo Joe, Hun Taeg Chung, Rina Yu

**Affiliations:** Department of Food Science and Nutrition, University of Ulsan, Ulsan, 680-749 South Korea; Department of Pharmacology and Dental Therapeutics, School of Dentistry, Chosun University, Gwangju, 501-759 South Korea; Graduate School of Agriculture, Kyoto University, Uji, Kyoto 611-0011 Japan; Department of Biological Sciences, University of Ulsan, Ulsan, 680-749 South Korea

**Keywords:** Obesity, Hepatosteatosis, Fatty liver disease, Mitochondrial oxidative metabolism

## Abstract

**Background:**

Obesity-induced hepatic lipid accumulation causes lipotoxicity, mitochondrial dysfunction, oxidative stress, and insulin resistance, and is implicated in non-alcoholic hepatic pathologies such as steatohepatitis and fibrosis. Heme oxygenase-1 (HO-1), an important antioxidant enzyme catalyzing the rate-limiting step in heme degradation, protects against oxidative stress, inflammation, and metabolic dysregulation. Here, we demonstrate that the phytochemical, quercetin, a natural polyphenol flavonoid, protects against hepatic steatosis in obese mice fed a high-fat diet, and that it does so by inducing HO-1 and stimulating increased hepatic mitochondrial oxidative metabolism.

**Methods:**

Male C57BL/6 mice were fed a regular diet (RD), a high-fat diet (HFD), and an HFD supplemented with quercetin for 9 weeks. Levels of mitochondrial biogenesis and oxidative metabolic transcripts/proteins were measured by real-time PCR and/or Western blotting. HO-1 transcripts/proteins were measured real-time PCR and/or Western blotting.

**Results:**

Quercetin upregulated genes involved in mitochondrial biogenesis and oxidative metabolism in lipid-laden hepatocytes and the livers of HFD-fed obese mice, and this was accompanied by increased levels of the transcription factor, nuclear erythroid 2-related factor 2 (Nrf-2), and HO-1 protein. The HO-1 inducer hemin and the HO-1 byproduct carbon monoxide (CO) also enhanced hepatic oxidative metabolism in HFD-fed obese mice. Moreover, the metabolic changes and the lipid-lowering effects of quercetin were completely blocked by the HO-1 inhibitor ZnPP and by deficiency of Nrf-2.

**Conclusion:**

These findings suggest that quercetin stimulates hepatic mitochondrial oxidative metabolism by inducing HO-1 via the Nrf-2 pathway. Quercetin may be useful in protecting against obesity-induced hepatosteatosis.

## Background

Obesity is a major risk factor for non-alcoholic fatty liver diseases (NAFLD), a condition ranging from excess triglyceride accumulation as lipid droplets within hepatocytes (fatty liver/steatosis) to steatohepatitis, which is accompanied by hepatocyte injury and inflammation, cell death, and fibrosis [1] and increases the risk of progression to cirrhosis as well as hepatocellular carcinoma [2]. Obesity-induced hepatic lipid accumulation results from uptake of circulating free fatty acids and *de novo* hepatic lipogenesis, leading to lipotoxicity, mitochondrial dysfunction, oxidative stress, and insulin resistance [1]. In addition, the reduction in mitochondrial oxidative metabolism leads to accumulation of partially oxidized intermediates, which further exacerbates insulin resistance and promotes the development of NAFLD [3, 4]. Hence, improving mitochondrial function and biogenesis in obese conditions might be beneficial in protecting against the development and progression of NAFLD.

Heme oxygenase-1 (HO-1), an important antioxidant enzyme catalyzing the rate-limiting step in heme degradation, is one of factors protecting against oxidative stress, inflammation, and metabolic dysregulation [5, 6]. HO-1 expression is regulated by the activation of nuclear factor erythroid 2-related factor 2 (Nrf-2), a basic leucine zipper transcription factor [7]. Carbon monoxide (CO), a byproduct of HO-1 activity increases mitochondrial biogenesis by binding to cytochrome c oxidase and stimulating mitochondrial ROS production [8]; it is therefore implicated in mitochondrial oxidative phosphorylation. Moreover, we and others have reported that HO-1 induction by hemin protects against obesity-induced metabolic dysregulation by reducing adipose inflammation [9], which causes mitochondrial dysfunction leading to loss of oxidative capacity and hepatic lipid accumulation [4, 10]. We therefore hypothesized that dietary factors that promote HO-1 induction and/or Nrf-2 activation might help to maintain hepatic mitochondrial oxidative capacity in obese conditions.

Quercetin (3, 3, 4, 5, 7-pentahydroxyflavone) is a flavonoid that is abundant in various fruits and vegetables and stimulates antioxidant and anti-inflammatory activities [11–14]. We previously showed that it decreased chemokine-induced adipose inflammatory responses by inhibiting the activation of inflammatory signaling molecules [15], and protected against obesity-induced skeletal muscle inflammation [16]. It also reduced ethanol-derived oxidative stress in human hepatocytes [17], and stimulated mitochondrial biogenesis in HepG2 cells and lipopolysaccharide-injected mice [18]. It has been suggested that its protective effect is due to HO-1 induction [18]. Quercetin also reduced the expression of genes encoding lipogenic enzymes in rat-liver cells and diet-induced obese mice [19, 20]. However, its effects on hepatic mitochondrial oxidative metabolism in obese condition remain unclear.

In this study, we demonstrate that quercetin reduces lipid accumulation in primary hepatocytes and the livers of obese mice fed a high-fat diet, and that this is associated with enhanced hepatic mitochondrial oxidative metabolism and HO-1 induction. The metabolic effects of quercetin were blocked by an HO-1 inhibitor and Nrf-2 deficiency. These findings indicate that the beneficial action of quercetin is associated with HO-1 induction through the Nrf-2 pathway. Quercetin may therefore be useful as a dietary additive for reducing obesity-induced hepatosteatosis.

## Materials and methods

### Animal experiment

All animal experiments were approved by the animal ethics committee of the University of Ulsan, and conformed to National Institutes of Health guidelines. Six-week-old male C57BL/6 mice were purchased from Orient Ltd. (Orient, Busan, Korea). The mice were maintained under specific pathogen-free conditions at 22 °C and given access to food and water ad libitum.

To examine the effects of quercetin on obesity-induced liver disease, we started the quercetin supplementation together with the establishment of diet-induced obesity. Mice (C57BL/6, male) were adapted for 1 week and then randomly divided into four dietary groups (*n* = 6 per group) and fed for 9 weeks on (1) a regular diet (RD) (3.1 kcal/g; 18 % calories from soybean oil; 58 % as carbohydrate; 24 % as protein; Harlan Teklad, Madison, WI); (2) a high-fat diet (HFD) (5.24 kcal/g; 60 % calories from lard and soybean oil; 20 % as carbohydrate; 20 % as protein; Research Diets, New Brunswick, NJ); (3) the HFD supplemented with 0.05 % (w/w) quercetin (HFD + 0.05 % Que); and (4) the HFD supplemented with 0.1 % quercetin (HFD + 0.1 % Que). The dosages of quercetin are equivalent to previous studies [16, 18], which are calculated to be ~50 and 100 mg/kg body weight per day, respectively.

To evaluate the effect of hemin on obesity-induced liver disease, mice were randomly assigned to the following experimental groups (*n* = 5 per group): (1) the RD + vehicle (RD), (2) the HFD + vehicle (HFD), and (3) the HFD + Hemin. Hemin (Sigma-Aldrich, St. Louis, MO) was dissolved in 0.15 M NaCl plus 10 % ammonium hydroxide (NH_4_OH) as a stock solution of 100 mg/mL and then further diluted 1 : 40 with sterile 0.15 M NaCl. The diluter was intraperitoneally injected (25 mg/kg BW) into the mice three times per week for 2 weeks [9, 21]. Vehicle-injected mice received an identical NH_4_OH-containing solution lacking hemin.

To evaluate the effect of CO, on obesity-induced liver disease, mice were randomly assigned to the following experimental groups (*n* = 6–7 per group): (1) the RD + air (RD), (2) the HFD + air (HFD), and (3) the HFD + carbon monoxide (HFD + CO). Mice inhaled CO (250 ppm) in air (Core Gas Ulsan, Korea) for 2 h (10 AM-12 PM) each day for 10 week. Mice were placed in an exposure chamber (LB science, Daejeon, Korea) at room temperature for exposure to air (control) or to 250 ppm CO as monitored by a CO probe (Tongoy Control Technology, Beijing, China) [22].

### Isolation of hepatocytes

Cultures of mouse hepatocytes were prepared as previously described [23]. Briefly, C57BL/6 male mice or Nrf-2 deficient mice (B6/SJL background), 6–8 weeks old, were anesthetized with Nembutal (Dainippon Sumitomo Pharma, Japan) intraperitonealy and their livers were perfused with 40 ml of Liver Perfusion Medium (Gibco, Grand Island, NY) followed by 30 ml of Liver Digestion Medium (Gibco), both at a flow rate of 5 ml/min. Hepatocytes were dispersed in Hepatocyte Wash Medium (Gibco) supplemented with 1 % penicillin/streptomycin by dissection and gentle shaking. After filtration through a 100 μm nylon mesh filter, hepatocytes were purified by repeated centrifugation (three times) at 50 × *g* for 2 min. A typical yield was about 4–5 × 10^7^ hepatocytes/mouse with >80 % cell viability as determined by trypan blue exclusion assay. The purity of hepatocytes was determined by flow cytometry (FACSCanto II, BD Bioscience, San Jose, CA). Hepatocytes and non-hepatocytes were separated based on cell size using forward scatter (FSC) and side scatter (SSC). Data were analyzed with Diva Software (BD Bioscience), and 85 % of the cells were found to be hepatocytes. The isolated hepatocytes were resuspended in DMEM (Gibco) supplemented with 10 % FBS, and 1 % penicillin/streptomycin, and cultured in type-1 collagen-coated 12-well plates at a cell density of 2 × 10^5^ cells/well.

### Cell culture and treatment of hepatocytes

To establish lipid-laden hepatocyte, mouse primary hepatocytes on 12-well plates (2 × 10^5^ cells) were treated with 1 mM FFA mixture, a 2:1 ratio of oleate/palmitate coupled to fatty acid-free BSA (molar ratio, 10:1) in DMEM supplemented with 10 % FBS, and 1 % penicillin/streptomycin for 24 h. The cells were exposed to various concentrations of quercetin and/or 0.1 μM ZnPP, an HO-1 inhibitor for 24 h incubation.

### Fasting glucose and insulin levels, and glucose tolerance tests

Plasma insulin levels were determined with the Ultrasensitive Mouse Insulin ELISA (Mercodia, Uppsala, Sweden), and glucose levels were determined with an Accu-Chek glucose monitor and test strips (Roche Diagnostics, Indianapolis, IN). For oral glucose tolerance tests, mice were fasted 12 h before receiving by oral administration of a 20 % glucose solution at a dose of 2 g/kg. Blood samples were taken from tail veins at before and 30, 60, 90, and 120 min after glucose administration and analyzed for glucose levels.

### Determination of lipid peroxidation

Hepatic lipid peroxidation levels were determined by measuring the levels of thiobarbituric acid-reactive substances (TBARS). Briefly, samples were mixed with TBA reagent consisting of thiobarbituric acid (TBA) and 15 % trichloroacetic acid in 0.25M HCl. The reaction mixture was boiled in a water bath for 1 h and centrifuged at 2000 rpm for 10 min. The TBARS concentration was determined at 520 nm absorbance with tetra-methoxypropane as standard. The protein content of homogenates was determined with a BCA protein assay kit (Pierce, Rockford, IL).

### Hepatic histology

Liver tissues were fixed overnight at room temperature in 10 % formaldehyde and embedded in paraffin. Eight micron thick sections were stained with hematoxylin-eosin and mounted on glass slides. Stained sections were viewed with an Axio-Star Plus microscope (original magnification ×200; Carl Zeiss, Gottingen, Germany).

### Hepatic lipid levels

Hepatic triglyceride content was assayed by saponification in ethanolic KOH, and glycerol content was measured with an FG0100 kit (Sigma-Aldrich) after neutralization with MgCl_2_. All tissue triglyceride values were converted to glycerol content and corrected for liver weight. Plasma alanine aminotransferase (ALT) level was measured using commercial kits (Asan Pharm. Co., LTD, Gyeonggi-do, Korea).

### Real-time PCR

Total RNA extracted from cultured cells was reverse transcribed into cDNA using M-MLV reverse transcriptase (Promega, Madison, WI). Real-time PCR amplification of the cDNA was performed in duplicate with a SYBR premix Ex Taq kit (TaKaRa Bio Inc., Foster, CA) using a Thermal Cycler Dice (TaKaRa Bio Inc., Japan). All reactions were performed by the same procedure: initial denaturation at 95 °C for 10 s, followed by 45 cycles of 95 °C for 5 s and 60 °C for 30 s. Results were analyzed with real-time system TP800 software (Takara Bio, Inc.) and all values for genes were normalized to values for a housekeeping gene. The primers used in the analysis are listed in Table 1.

**Table 1:** Mouse primers used for real-time PCR analysis

Gene	Forward primer (5′ → 3′)	Reverse primer (5′ → 3′)
Nrf-2	TCCGCTGCCATCAGTCAGTC	ATTGTGCCTTCAGCGTGCTTC
HO-1	TGCAGGTGATGCTGACAGAGG	GGGATGAGCTAGTGCTGATCTGG
PGC-1α	CCGTAAATCTGCGGGATGATG	CAGTTTCGTTCGACCTGCGTAA
Nrf-1	GACCTTGCCACAGGCAGGTAA	CGCCTGCTCCATGAACACTC
Tfam	TCAGGAGCAGCAGGCACTACA	CTGAGCTCCGAGTCCTTGAACAC
PPARα	ACGCTCCCGACCCATCTTTAG	TCCATAAATCGGCACCAGGAA
CPT-1α	TGGCTTCAGAGCCAGTGGAG	AGCGATGGTGGCTGTCATTC
β-actin	CATCCGTAAAGACCTCTATGCCAAC	ATGGAGCCACCGATCCACA
GAPDH	GGCTATCACGGAGGCTGTGAA	CCAGCCTTAGCATCAAAGATGGA

### Western blot analysis

Samples of 10 ~ 50 μg total protein were subjected to western blot analysis using polyclonal antibodies to phosphorylated Akt (Akt-pSer^473^), Akt (Cell Signaling, Beverly, MA), HO-1 (Enzo Life Sciences, Farmingdale, NY), COX IV (Abcam, Cambridge, MA), α-Tubulin (Abcam), and β-actin (Sigma-Aldrich). Protein bands were detected using an enhanced chemiluminescence Western blotting detection kit (PerkinElmer, Waltham, MA). Band intensities were quantified by densitometry using Image J program.

### Statistical analyses

Results are presented as means ± SEM. Statistical analyses were performed using Student’s *t* test. Differences were considered to be significant at *p* < 0.05.

## Results

### Effect of quercetin on hepatic steatosis and glucose intolerance in obese mice fed an HFD

**Fig. 1:**
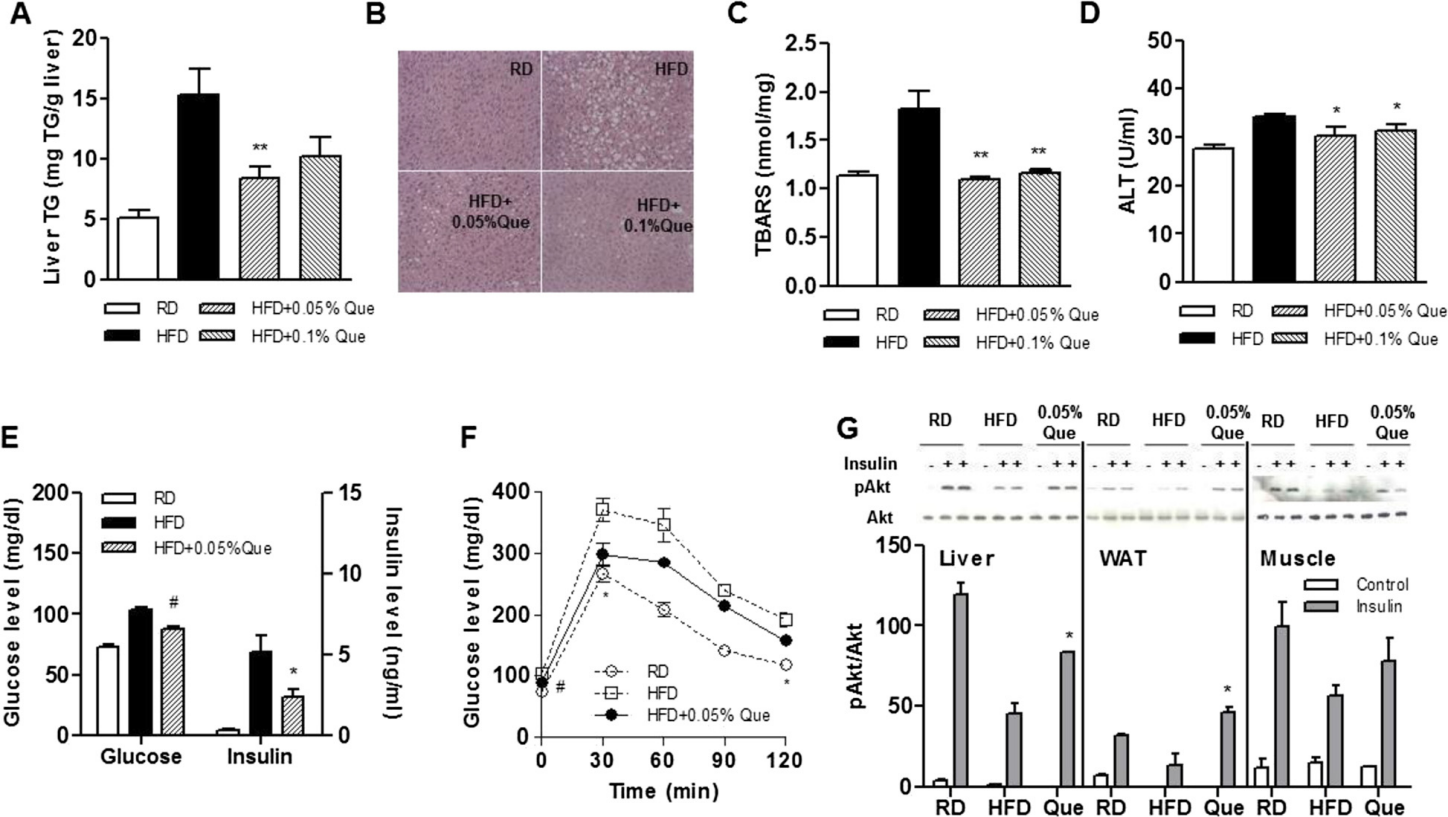
Effect of quercetin on hepatic lipid accumulation and glucose tolerance in HFD-fed obese mice. C57BL/6 mice were fed a regular diet (RD), a high-fat diet (HFD), an HFD supplemented with 0.05 % quercetin (HF + 0.05 % Que) or 0.1 % quercetin (HFD + 0.1 % Que) for 9 weeks (*n* = 6 per group). **a** Liver tissues were collected and their TG content was determined. **b** Representative images of hematoxylin and eosin stained tissue. Original magnification, 200 ×. **c** Hepatic TBARS levels as a marker of lipid peroxidation. **d** Plasma alanine aminotransferase (ALT) levels. Data are mean ± SEM of six mice per group. *p < 0.05, **p < 0.01 versus HFD. **e** Fasting glucose and insulin levels. **f** Oral glucose tolerance test. Mice were fasted 12 h before receiving by mouth a 20 % glucose solution at a dose of 2 g/kg, and blood samples were taken at the indicated times. Levels of glucose were measured using the glucometer. Data are mean ± SEM of six mice per group. *p < 0.05, # *p* < 0.005 versus HFD. **g** Insulin responses. After fasting 5 h, mice were stimulated with or without insulin for 4 min. Expression of p-Akt, and Akt proteins in the liver, adipose tissue, and skeletal muscle of each mouse (*n* = 4 per group) were examined by Western blot analysis using the indicated antibodies. Data are mean ± SEM of four mice per group. *p < 0.05 versus HFD

To examine the effects of quercetin in vivo, we generated obese mice fed an HFD with or without quercetin. Quercetin supplementation significantly reduced hepatic triglyceride concentration in the liver of the HFD-fed obese mice (Fig. 1a). In agreement with this, lipid deposition in the liver revealed by histochemical analysis was reduced (Fig. 1b). Quercetin also significantly reduced TBARS levels, a marker of lipid peroxidation (Fig. 1c), and ALT levels, a marker of liver damage (Fig. 1d). We further examined whether the metabolic improvement in response to quercetin observed in the livers of the obese mice reduced obesity-induced glucose intolerance. Levels of fasting glucose and insulin were significantly lower by quercetin (Fig.1e). Glucose tolerance tests confirmed that the quercetin-fed obese mice were more glucose tolerant (Fig. 1f), and activation of the insulin signaling molecules Akt in liver, muscle, and adipose tissue was stimulated (Fig. 1g). Quercetin did not alter food intake, and the HFD-fed mice supplemented with quercetin had a tendency to gain less weight than the control HFD-fed mice (data not shown), as previously reported [16], indicating that the observed benefits of quercetin supplementation are probably associated with the metabolic effects of quercetin.

### Induction of HO-1 by quercetin in liver of obese mice fed an HFD

**Fig. 2:**
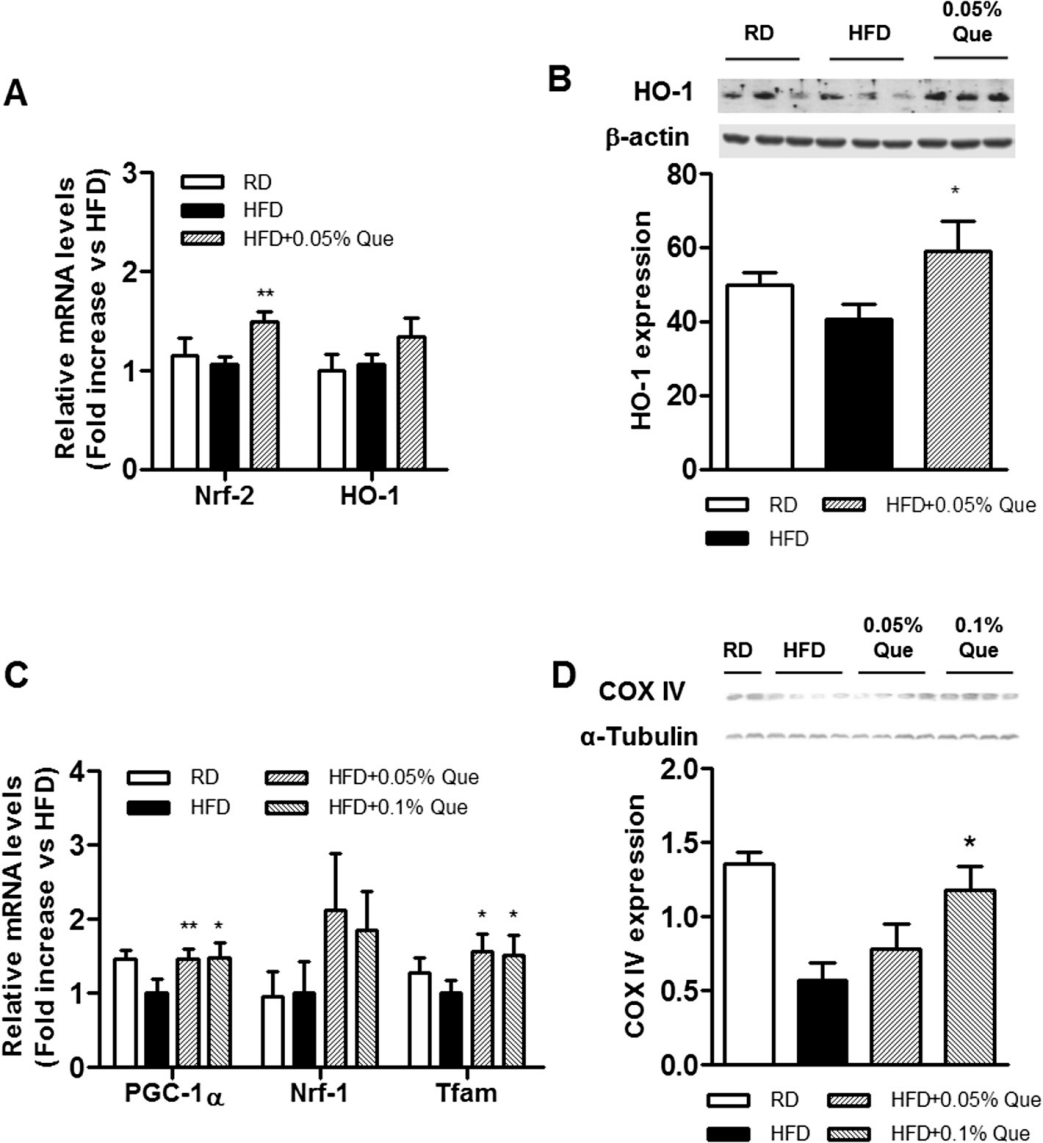
Effect of quercetin on expression of HO-1 and/or mitochondrial biogenesis markers in HFD-fed obese mice. Livers were isolated from mice fed a regular diet (RD), a high-fat diet (HFD), an HFD supplemented with 0.05 % quercetin (HF + 0.05 % Que) or 0.1 % quercetin (HFD + 0.1 % Que) for 9 weeks (*n* = 6 per group). **a** Expression of Nrf-2 and HO-1 mRNAs was quantified by real-time PCR. **b** Levels of HO-1 protein were determined by Western blotting. **c** Expression of PGC-1α, Nrf-1, and Tfam mRNAs was quantified by real-time PCR. **d** Levels of COX IV protein were determined by Western blotting. Data are mean ± SEM of six mice per group. *p < 0.05, **p < 0.01 versus HFD

HO-1 plays an important role in modulating hepatic metabolism by affecting mitochondrial biogenesis and metabolism [24, 25]. We found that quercetin supplementation significantly upregulated levels of Nrf-2 and HO-1 transcripts (Fig. 2a), and also increased HO-1 protein in the HFD-fed obese mice (Fig. 2b). To see whether it altered mitochondrial oxidative metabolism, we measured markers for mitochondrial biogenesis and oxidative phosphorylation. Levels of PGC-1α and Tfam transcripts (Fig. 2c), and COX IV protein (Fig. 2d) were indeed also upregulated.

### HO-1 inhibition and Nrf-2 deficiency oppose the effects of quercetin

**Fig. 3:**
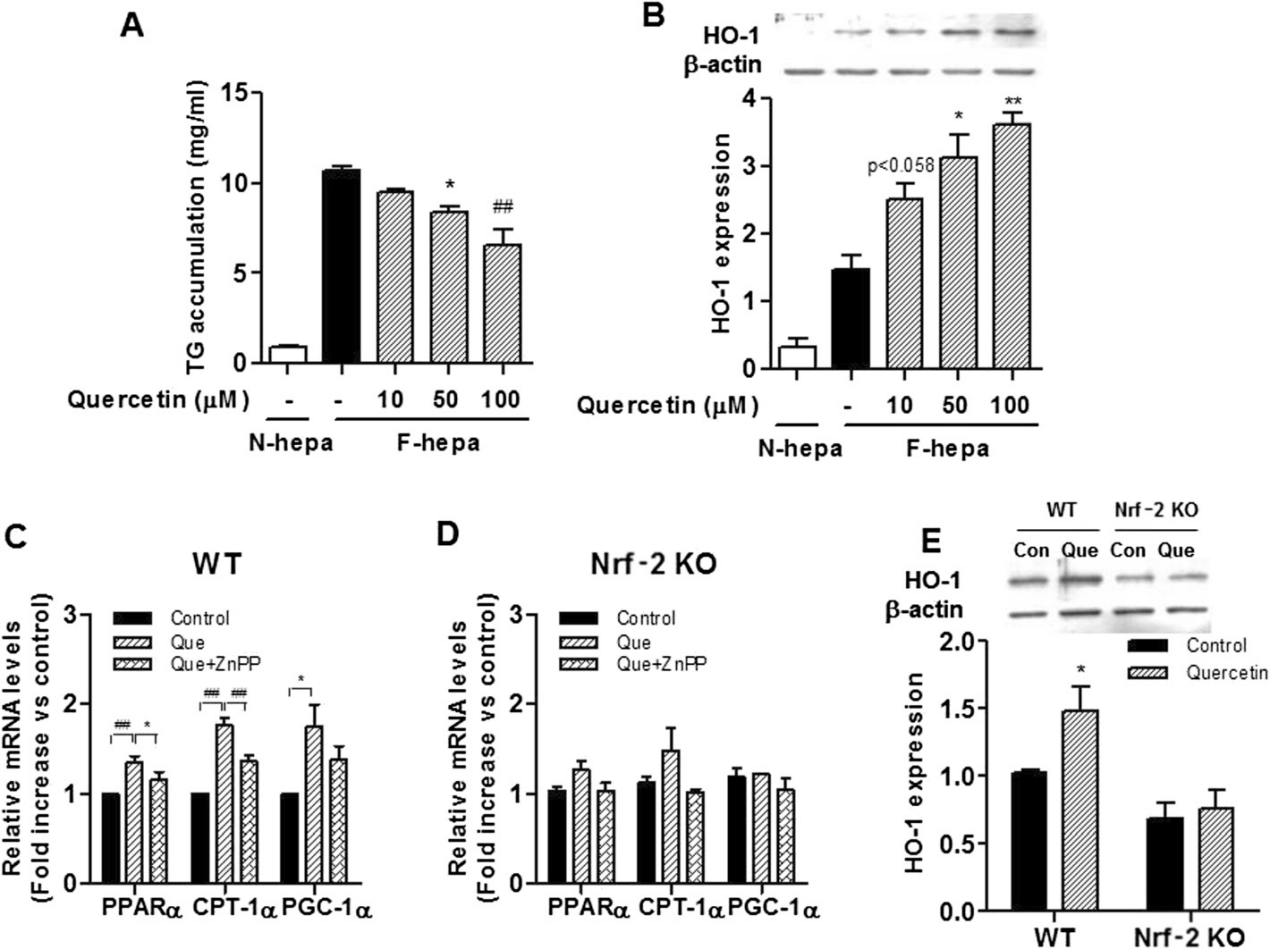
Effects of quercetin on lipid accumulation and HO-1 induction in hepatocytes. **a**-**b** Mouse primary hepatocytes were incubated with a 1 mM FFA mixture (2:1 ratio of oleate/palmitate) for 24 h, and exposed to various concentrations of quercetin. N-hepa was added with 100 μM BSA. N-hepa; normal hepatocyte, F-hepa; fatty hepatocytes. **a** Intracellular TG content. Data are mean ± SEM of quadruplicate samples. *p < 0.05, ## *p* < 0.001 versus F-hepa. **b** HO-1 protein was determined by Western blotting. Data are mean ± SEM from two independent experiments. *p < 0.05, **p<0.01 versus F-hepa. **c**-**d** Mouse primary hepatocytes from Nrf-2 deficient and WT mice were incubated with 1 mM FFA mixture (2:1 ratio of oleate/palmitate) for 24 h and exposed 10 μM quercetin and/or 0.1 μM ZnPP, an HO-1 inhibitor. Control cells were incubated with FFA mixture. **c** Wild type lipid-laden hepatocytes. **d** Nrf-2 deficient lipid-laden hepatocytes. Expressions of mRNAs associated with lipid oxidation (PPARα and CPT-1α) and mitochondrial biogenesis (PGC-1α) were quantified by real-time PCR. Data are mean ± SEM from three independent experiments. *p < 0.05, ## *p* < 0.001 versus Que. **e** HO-1 protein was determined by Western blotting. Data are mean ± SEM from four independent experiments. *p < 0.05 versus WT-Con

Using lipid-laden primary hepatocytes mimicking fatty hepatocytes, we tested the idea that quercetin reduces hepatic lipid accumulation by inducing HO-1. As shown in Fig. 3a and b, the effect of quercetin on the lipid content of lipid-laden hepatocytes was paralleled by an increase in the level of HO-1 protein. Along with this we found that markers of mitochondrial biogenesis such as PGC-1α, and PPARα and CTP-1α, a marker of mitochondrial oxidative phosphorylation, were upregulated (Fig. 3c). Moreover, ZnPP, a competitive inhibitor of HO-1, significantly reduced these effects (Fig. 3c), and the effects were completely abolished in Nrf-2 deficiency (Fig. 3d). Moreover, quercetin also had no effect on HO-1 protein levels in the Nrf-2-deficient hepatocytes, while it markedly induced HO-1 expression in the WT control (Fig. 3e).

### Effect of hemin and carbon monoxide on hepatic mitochondrial metabolism

**Fig. 4:**
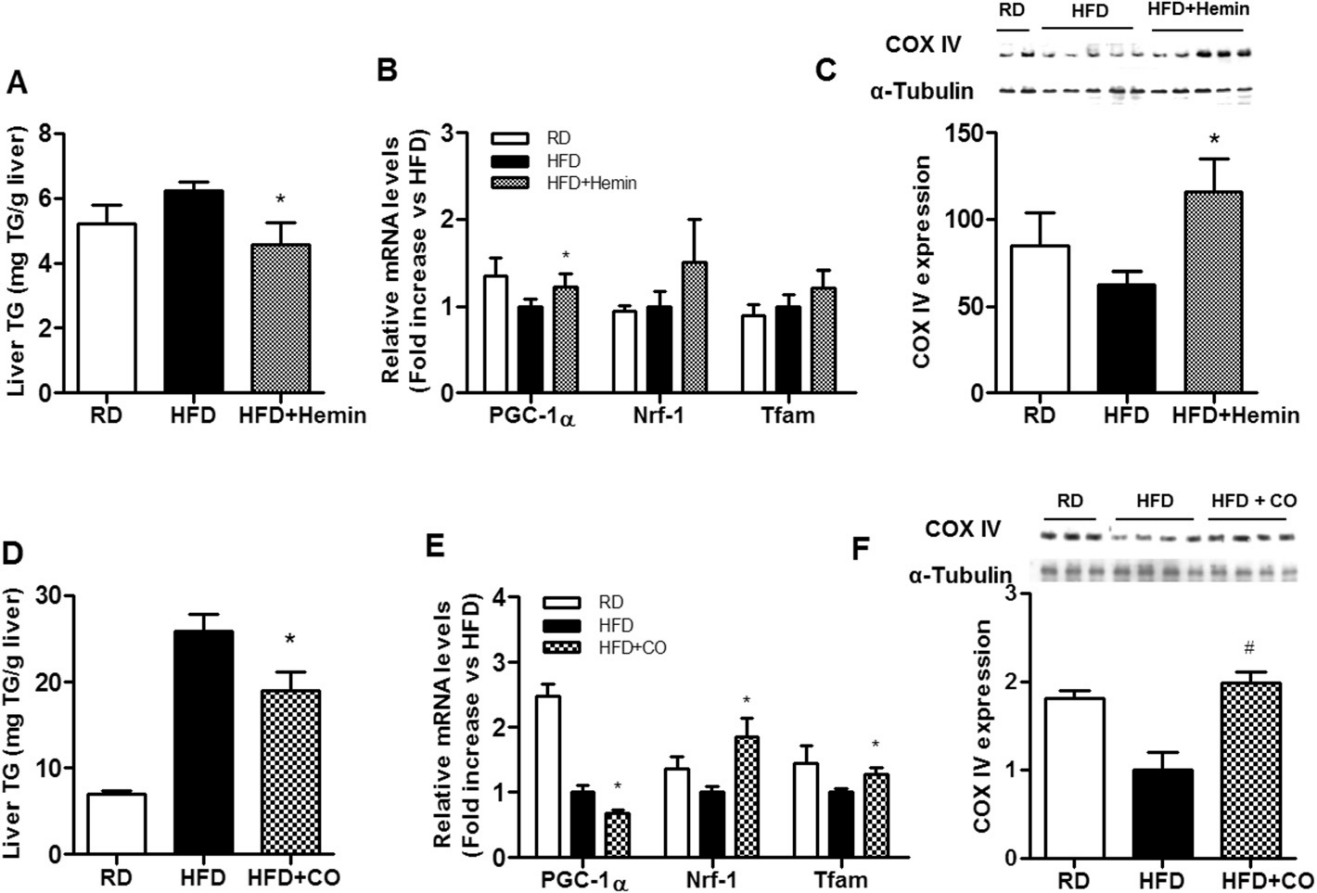
Effect of an HO-1 inducer and/or CO on hepatic lipid accumulation and mitochondrial oxidative metabolism in HFD-fed mice (**a**-**c**) C57BL/6 mice were fed an HFD diet for 2 weeks with hemin injection three times per week (*n* = 5 per group). **a** Liver tissues were collected and their TG contents were determined. **b** PGC-1α, Nrf-1, and Tfam mRNAs were quantified by real-time PCR. **c** Expression levels of COX IV protein was determined by Western blotting. Data are mean ± SEM of five mice per group. *p < 0.05 versus HFD. **d**-**f** C57BL/6 mice inhaled CO (250 ppm) for 2 h each day for 10 weeks (*n* = 6–7 per group). **d** Liver tissues were collected and their TG contents were determined. **e** PGC-1α, Nrf-1, and Tfam mRNAs were quantified by real-time PCR. **f** Expression levels of COX IV protein were determined by Western blotting. Data are mean ± SEM of six to seven mice per group. *p < 0.05, # *p* < 0.005 versus HFD

We also confirmed the effects of the HO-1 inducer hemin and the HO-1 byproduct carbon monoxide on hepatic lipid content in HFD-fed obese mice. Like quercetin, both treatments reduced hepatic lipid accumulation (Fig. 4a and d) and enhanced markers of mitochondria biogenesis such as Tfam and PGC-1α transcripts (Fig. 4b and e) as well as a marker of oxidative metabolism, COX IV protein (Fig. 4c and f) in the livers of HFD-fed obese mice.

## Discussion

The uptake of circulating free fatty acids together with *de novo* hepatic lipogenesis can lead to harmful hepatic lipid accumulation. Hence, identifying dietary factors that reduce hepatic lipogenesis and/or increase hepatic lipid oxidation may be useful in reducing hepatic lipid accumulation in obese condition. Quercetin reduces the biosynthesis of hepatic fatty acids and triglycerides in the liver of HFD-fed mice [19]. In this study, we showed that quercetin enhances hepatic mitochondrial oxidative metabolism in lipid-laden hepatocytes and the livers of obese mice fed a high-fat diet, and for the first time demonstrated a causal relationship between the expression of Nrf-2/HO-1, mitochondrial biogenesis and hepatic lipid accumulation using hemin, ZnPP and CO.

Upregulation of transcripts and proteins involved in mitochondrial biogenesis leads to enhancement of oxidative metabolic capacity. For example, the transcription factor Nrf-1 regulates the transcription of nuclear genes coding for mitochondrial respiratory chain proteins, and it can be activated by PGC-1α, which stimulates the transcription of genes involved in oxidative phosphorylation [26, 27]. PGC-1α and Nrf-1 coactivate the expression of Tfam, which is important for regulating and maintaining mtDNA replication and transcription [26, 27]. Moreover, increases of COX IV typically reflect increased levels of other mitochondrial enzymes of the electron transport chain and enzymes of the *β*-oxidation pathway [28]. In this study, we found that quercetin increased transcript levels of genes involved in regulating mitochondria biogenesis such as PGC-1α, Nrf-1, Tfam, as well as COX IV protein in HFD-fed obese mice and in lipid-laden hepatocytes. Others have shown that obesity-induced reduction of transcript of PPARα, which regulates *β*-oxidation, is prevented by quercetin supplementation [19, 29]. Our findings together with these others suggest that quercetin improves hepatic mitochondrial oxidative metabolic capacity by increasing mitochondria biogenesis, and so limits hepatic lipid accumulation. Alternatively, quercetin is a potent scavenger of ROS in many types of cells [30–32], and this may contribute to reduction of mitochondrial oxidative damage and hence maintain its oxidative metabolic capacity. In addition, the reductions in TBARS and plasma levels of ALT indicate that quercetin protects against hepatic mitochondrial damage, which may rescue mitochondrial oxidative capacity.

**Fig. 5:**
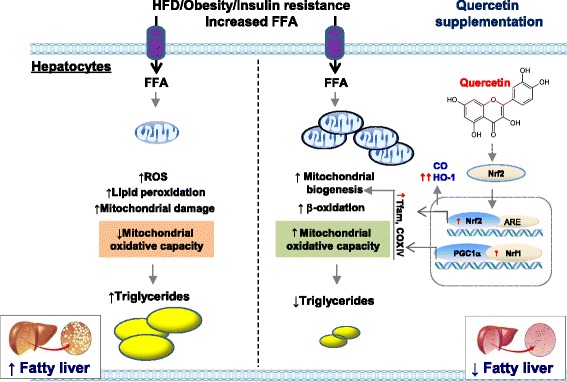
Proposed mechanism for quercetin action against hepatic steatosis in obese mice. Excess intake of nutrients, including overloaded FFAs in obese conditions, increases ROS production in the liver; the latter results from lipid peroxidation and cause mitochondrial damage. The mitochondrial damage in turn leads to a decrease of mitochondrial oxidative capacity, including β-oxidation and ATP production, resulting in triglyceride accumulation in the liver. Quercetin limits hepatic lipid accumulation by enhancing hepatic mitochondrial oxidative metabolic capacity. Quercetin increases the induction of HO-1 and its byproduct CO by the upregulated transcriptional regulator Nrf-2. CO, enhances the nuclear translocation of Nrf-1 and PGC-1α, and activates Tfam expression, all of which augments mitochondrial biogenesis. This leads to reduction of FFA-induced lipid peroxidation, mitochondrial damage and hepatic triglyceride accumulation by increasing mitochondrial oxidative capacity. The effects of quercetin on the induction of HO-1 were completely disrupted in HO-1 inhibitor and/or Nrf-2 deficiency, indicating that one of the mechanisms by which quercetin enhances mitochondrial oxidative metabolism is associated with HO-1 induction through Nrf-2 pathway

HO-1 metabolizes heme to biliverdin and releases carbon monoxide and iron [33], and plays an important role in cellular defense against oxidative damage and in cellular metabolism [25]. The transcription factor Nrf-2 is the master regulator of HO-1 gene expression, and the HO-1 product, carbon monoxide, enhances the nuclear translocation of Nrf-1 and PGC-1α, and activates Tfam expression, all of which augments mitochondrial biogenesis [34, 35]. Studies have shown that quercetin attenuates hepatic lipid accumulation in obese mice fed an HFD [19, 31, 32] and that reduced lipid accumulation is associated with Nrf-2 [31]. Quercetin also increases hepatic mitochondrial biogenesis [18]. However, the link between hepatic lipid accumulation and mitochondrial biogenesis and Nrf-2/HO-1 signaling remained unclear. Interestingly, we observed upregulation of HO-1 protein in the livers of obese mice supplemented with quercetin and in lipid-laden hepatocytes treated with quercetin. Moreover, we found that the HO-1 inducer hemin and the HO-1 byproduct carbon monoxide enhanced levels of transcripts involved in mitochondria biogenesis and oxidative phosphorylation, and reduced hepatic lipid accumulation, indicating that the effect of quercetin may be due to HO-1 induction. Moreover the beneficial metabolic effect of quercetin on hepatic mitochondrial metabolic markers was abrogated by the HO-1 inhibitor ZnPP in lipid-laden hepatocytes. Quercetin also upregulated Nrf-2, a transcriptional regulator of HO-1, suggesting that quercetin-induced HO-1 upregulation is mediated by the Nrf-2 pathway. Indeed, we found that the effects of quercetin on the induction of HO-1 were completely disrupted in Nrf-2 deficient lipid-laden hepatocytes, and this was accompanied by a reduction in mitochondrial oxidative metabolic transcripts. These results suggest that one of the mechanisms by which quercetin enhances mitochondrial oxidative metabolism is associated with HO-1 induction through Nrf-2 pathway (Fig. 5).

## Conclusion

In conclusion, quercetin reduced obesity-induced hepatic lipid accumulation by enhancing mitochondrial oxidative capacity, and this was accompanied by induction of HO-1. The action of quercetin was blunted by HO-1 inhibitor and Nrf-2 deficiency, indicating that HO-1 induction by quercetin through Nrf-2 pathway may be among mechanisms contributing to the reduction of obesity-induced hepatic lipid accumulation. Quercetin, an inducer of HO-1, may be useful as a dietary factor for reducing obesity-induced hepatosteatosis.

## Abbreviations

NAFLD: Non-alcoholic fatty liver diseases
Que: Quercetin
RD: Regular diet
HFD: High-fat diet
ALT: Alanine aminotransferase
TBARS: Thiobarbituric acid-reactive substances
HO-1: Heme oxygenase-1
Nrf-2: Nuclear erythroid 2-related factor 2
PPARα: Peroxisome proliferator-activated receptor alpha
PGC-1α: Peroxisome proliferator-activated receptor gamma coactivator-1-alpha
CPT-1α: Carnitine palmitoyltransferase-1-alpha
Nrf-1: Nuclear respiratory factor 1
Tfam: Transcription factor A, mitochondrial
COX IV: Cytochrome C oxidase subunit 4
CO: Carbon monoxide
